# Downregulation of hsa-miR-100-5p May Be a Protective Factor in the Early Stages of Nephropathy in Type 1 Diabetes Mellitus

**DOI:** 10.3390/ijms25115663

**Published:** 2024-05-23

**Authors:** Andrey Henrique Gama Pinheiro, Beatriz de Oliveira Pereira, Lilian Souza D’Albuquerque Silva, Franciane T. Cunha de Melo, Ana Carolina C. Braga de Souza, Valéria S. Galvão Leal, Priscila B. Barbosa de Figueiredo, João F. Abrahão Neto, Marcia Costa dos Santos, Natércia Neves Marques de Queiroz, Karem Miléo Felício, Ândrea Ribeiro-dos-Santos, João Soares Felício, Giovanna C. Cavalcante

**Affiliations:** 1 Laboratory of Human and Medical Genetics, Graduate Program in Genetics and Molecular Biology, Federal University of Pará, Belém 66075-110, PA, Brazil; andrey.biomed@gmail.com (A.H.G.P.); oliverbibia@gmail.com (B.d.O.P.); akelyufpa@gmail.com (Â.R.-d.-S.); 2 Endocrinology and Metabology/Diabetes Unit, João de Barros Barreto University Hospital, Federal University of Pará, Belém 66075-110, PA, Brazil; liliandalbuquerque@gmail.com (L.S.D.S.); franciane.melo@hotmail.com (F.T.C.d.M.); carol.souza25@hotmail.com (A.C.C.B.d.S.); valeriasuenya@gmail.com (V.S.G.L.); pribfigueiredo@gmail.com (P.B.B.d.F.); jfabrahao2018@gmail.com (J.F.A.N.); marciacsantos29@gmail.com (M.C.d.S.); natercianeves@hotmail.com (N.N.M.d.Q.); karemfelicio@yahoo.com.br (K.M.F.)

**Keywords:** diabetic kidney disease, diabetic nephropathy, type 1 diabetes mellitus, miRNAs, hsa-miR-100-5p

## Abstract

Type 1 Diabetes Mellitus (T1DM) can generate severe complications, such as Diabetic Kidney Disease (DKD) or Diabetic Nephropathy (DN), with it emerging as the leading cause of terminal (end-stage) renal disease all over the world. For T1DM, the clinical evaluation of DKD uses markers like the Glomerular Filtration Rate (GFR) and the Urinary Albumin Excretion (UAE). However, early diagnosis of DKD is still a challenge. For this reason, investigating molecular markers, such as microRNAs (miRNAs), offers a promising perspective to an early diagnosis, highlighting the stability and the ability to reflect incipient molecular manifestations. Thus, here we investigated four miRNAs (hsa-let-7i-5p, hsa-miR-143-3p, hsa-miR-501-3p, and hsa-miR-100-5p) regarding nephropathy in patients with T1DM, considering the albuminuria (micro and macro) as a standard to evaluate the groups. As a result, we found a reduced expression of miR-100-5p in patients with MIC, indicating a protective role in nephropathy. Beyond that, expression levels between the groups (Non vs. UAE) were not significant when comparing the miRNAs miR-501-3p and miR-143-3p. Finally, miR-143-3p and miR-100-5p were linked to some target genes such as AKT1, MMP13, and IGF1R, that are connected to signal pathways and cellular metabolism.

## 1. Introduction

Type 1 Diabetes Mellitus (T1DM) is the resulting condition of the autoimmune destruction of beta-pancreatic cells, which are responsible for insulin production. This condition increases blood glucose, causing alterations in various body systems, including cardiovascular and renal. These complications are not T1DM exclusive, but common to Type 2 Diabetes Mellitus (T2DM), and both are associated with molecular and metabolic changes due to the syndrome [1,2,3].

**Figure 1:**
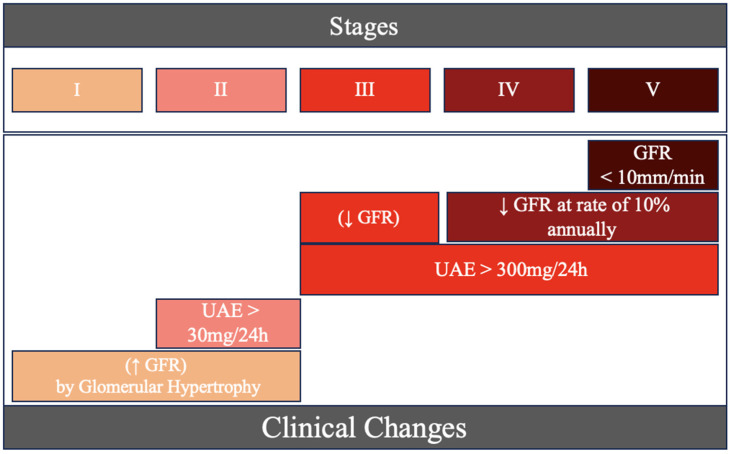
Five stages (I–V) of Diabetic Nephropathy’s clinical evolution.

One of the most significant complications of diabetes is Diabetic Nephropathy (DN) or Diabetic Kidney Disease (DKD), a form of Chronic Kidney Disease (CKD) [4,5]. We can observe this medical condition’s clinical evolution in Figure 1. These events result from diverse structural molecular and cellular alterations, such as the epithelial–mesenchymal transition, glomerular hypertrophy, and fibrosis, all contributing to the progressive loss of kidney function [6]. Diabetic Kidney Disease (DKD) emerges as the leading cause of terminal renal disease in the world [7]. This complication significantly impacts 20 to 40% of patients diagnosed with Diabetes Mellitus (DM) [8].

Triage protocols can change according to the type of diabetes diagnosed. For each patient with T2DM, the recommendation is to begin screening right after the diagnosis. Patients with T1DM should undergo it for five years after the diagnosis. It is crucial to highlight that there are specific protocols for teenagers, those in the pubertal phase, and individuals with uncontrolled glycemic levels; the screening must be conducted as soon as possible, and annually [5]. DKD’s clinical evaluation is based on specific diagnostic criteria. Glomerular Filtration Rate (GFR) values of less than 60 mL/min/1.73 m and Urinary Albumin Excretion (UAE) with a sustained increase over a minimum period of three months are considered indicators of significant renal impairment. The increased UAE can be defined by the albumin quantification in 24 h urine, with values higher than 30 mg/24 h, or by an albumin/creatinine ratio (ACR) equal to or greater than 30 mg/g [4,7,9]. 

The continuous monitoring of GFR, combined with the measurement of UAE, provides not only the detection of DKD but also contributes to categorizing the disease into specific stages. This structured approach, with patient stratification in stages, enables the definition of appropriate therapeutic measures, tailored to the specific needs of each stage of DKD [9]. Despite UAE and GFR being widely used to monitor patients with DKD, these methods have limitations, especially regarding early diagnosis, because observed clinical manifestations already represent the consequences of alterations in renal physiology, so the early identification of DKD remains a challenge [10]. 

Therefore, studies have concentrated on identifying molecular markers that could improve DKD diagnosis. A notable example of this approach is the investigation of microRNAs (miRNAs), non-coding RNAs (ncRNAs) composed of approximately 20–22 nucleotides, which play a crucial role in regulating gene expression at the post-transcriptional level. These molecules perform their function by binding to mRNA’s 3’UTR region, resulting in the suppression of translation or their degradation [11]. 

Investigating molecular markers such as miRNAs presents a promising prospect in mitigating the restrictions associated with conventional diagnostic methods and enabling the early identification of Diabetic Kidney Disease (DKD). This is based on the intrinsic stability of these miRNAs, which can reflect manifestations at molecular levels that are still incipient [9,12]. Several research groups have identified miRNAs differentially expressed in patients with and without DKD, using biological samples such as serum and urine [6,9,13]. However, due to the heterogeneity of the population, these studies have faced challenges in reproducibility in different cohorts.

Thus, it is crucial to identify specific molecular signatures that can improve early diagnosis, especially in people from the North of Brazil, where genetic and environmental characteristics can markedly vary if compared with the rest of the country and the world [14]. This personalized approach can contribute to a more effective diagnosis and a therapeutic plan of action’s implementation in the early management of DKD. Here, considering a previous study from our research group [15], we investigated four miRNAs (hsa-let-7i-5p, hsa-miR-143-3p, hsa-miR-501-3p e hsa-miR-100-5p) and their association with nephropathy in patients with T1DM from the North of Brazil. Additionally, other studies identified these five miRNAs, possibly involved in DRC, caused by T2DM or other diseases [16,17,18,19].

## 2. Results

### 2.1. Sample Characterization

After analyzing the clinical variables, we observed that there was a significant change between the clinical analytes studied related to DKD, according to the classification of albuminuria: normoalbuminuria or non-UAE (Non, n = 12), microalbuminuria (MIC, n = 4) and macroalbuminuria (MAC, n = 3). Table 1 shows the clinical characteristics of each studied group.

### 2.2. Characterization of miRNAs

**Figure 2:**
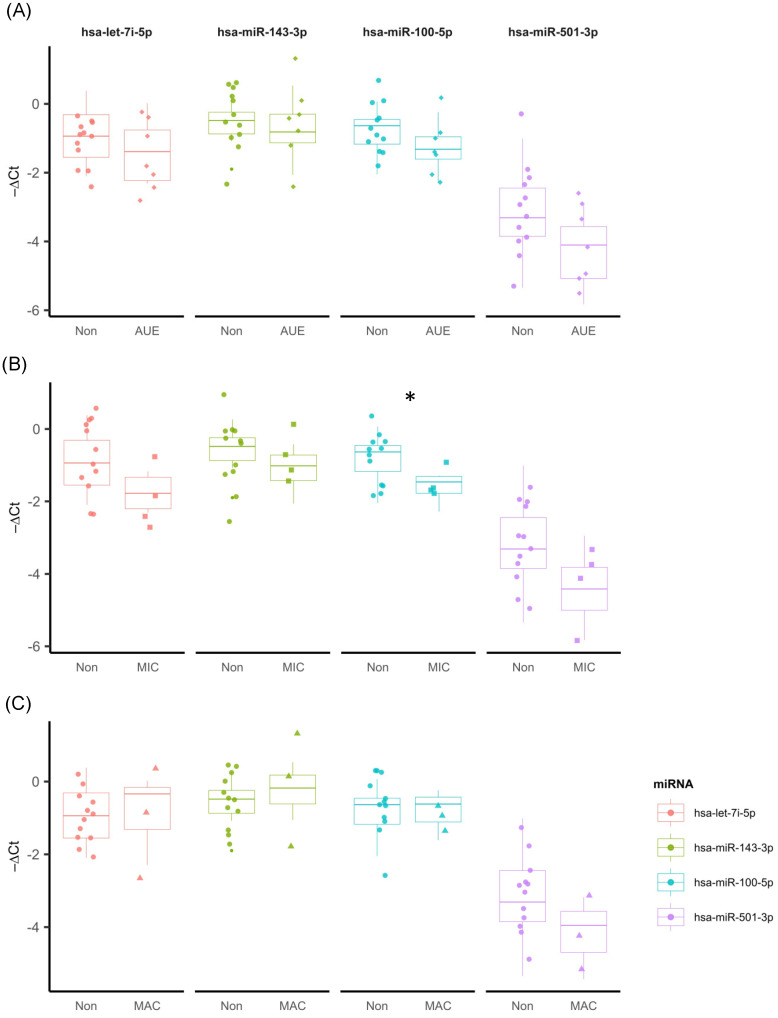
Expression profiles of the four miRNAs in three different conditions. (**A**) Non AUE (n = 12) and AUE (n = 7) patients: hsa-let-7i-5p (*p* = 0.299), hsa-mir-143-3p (*p* = 0.592), hsa-mir-501-3p (*p* = 0.068), and hsa-mir-100-5p (*p* = 0.142). (**B**) Comparison of −ΔCT values between Non (n = 12) and MIC (n = 4): hsa-let-7i-5p (*p* = 0.078), hsa-mir-143-3p (*p* = 0.133), hsa-mir-501-3p (*p* = 0.133), and hsa-mir-100-5p (*p* = 0.030) *. (**C**) Comparison of the average −ΔCT values between the Non (n = 12) and MAC (n = 3) groups for the miRNAs: hsa-let-7i-5p (*p* = 0.840), hsa-mir-143-3p (*p* = 0.448), hsa-mir-501-3p (*p* = 0.233), and hsa-mir-100-5p (*p* = 1.000).

We performed an analysis of four (let-7i-5p, mir-143-3p, mir-501-3p, mir-100-5p) of the five miRNAs, identified by Ferraz et al. [15] in the context of albuminuria in Diabetic Nephropathy. Figure 2A shows the relation between the ΔCT of the analyzed miRNAs; “ΔCt” represents the difference in cycle threshold (Ct) values between the endogenous reference gene and the target gene of interest. Although we had observed differences in the ΔCT rate of these miRNAs between the Non, MIC, and MAC groups, they were not statistically significant in all cases.

Figure 2B shows the relation between the ΔCT values of miRNAs hsa-let-7i-5p (*p* = 0.078), hsa-mir-143-3p (*p* = 0.133), hsa-mir-501-3p (*p* = 0.133) and hsa-mir-100-5p (*p* = 0.030) in individuals with microalbuminuria (MIC) and normoalbuminuria (Non). We observed that mean values of ΔCT for hsa-mir-100-5p were significantly higher in the MIC group when compared to the Non group, indicating the high expression of these miRNAs in the Non group. Figure 2C shows the comparison between the ΔCT values of these four miRNAs for Non and MAC groups; however, they were not statistically significant in all cases.

## 3. Discussion

### 3.1. hsa-let-7i-5p

Three of these miRNAs were previously highlighted by other researchers, considering the identification of molecular biomarkers linked to albuminuria. In a context related to DKD in T2DM patients and other complications, Prabu et al. compared Non-UAE T2DM vs. MAC T2DM and observed the hyperregulation of hsa-let-7i-5p in extracellular vesicles present in the urine of patients [16]. In addition, the same authors identified four miRNAs (hsa-let-7i-5p, miR-15b-5p, miR-24-3p, miR-27b-3p) that together compose a molecular signature able to distinguish Non-UAE T2DM patients from UAE (MIC/MAC) patients, showing promising levels of sensitivity and specificity, with an AUROC above 85%—the AUROC (Area under the ROC Curve) is a performance metric that evaluates the discrimination of a model. An AUROC of 85% means that the model has a good discriminatory ability: 85% of the time, the model will correctly assign a higher absolute risk to a randomly selected patient with an event (DM UAE) than to a randomly selected patient without an event (DM Non-UAE) [20]. An AUROC higher than 85% represents good performance [21] and demonstrates that, to some extent, hsa-let-7i-5p contributes to a miRNA signature present in individuals with DKD, suggesting a possible involvement of this miRNA in this pathological process.

Following the analysis, when the authors investigated the target genes associated with these miRNAs and their biological interaction, three protein networks were identified: one involving the Wnt/β-catenin signaling cascade, one involving activin receptor signaling, and the last involving the cell differentiation and proliferation [16].

The Wnt/β-catenin signaling pathway plays an essential role in the modulation of cellular proliferation, differentiation, and organ development, including the kidney. Its inappropriate activation has been linked to a variety of kidney disorders, including DKD. Similarly, activin receptor signaling is related to the cell’s growth regulation, differentiation, and immune response, and it is also associated with kidney disorders such as renal fibrosis. Beyond that, the regulation of cell differentiation and proliferation plays a crucial role in the pathophysiology of DKD, since cell dysfunction and uncontrolled proliferation can contribute significantly to the progression of kidney disease [22,23,24].

### 3.2. hsa-miR-143-3p

Additionally, in individuals diagnosed with arterial hypertension and CKD, a study conducted by Perez-Hernandez and colleagues [17] observed an upregulation of miR-143-3p, especially in urinary exosomes. It is suggested that miR-143-3p is one of the molecular components implicated in the loss of plasma proteins. This intriguing molecular mechanism of plasma protein loss can occur due to factors both external and internal to renal anatomy and physiology [25]. A promising mechanism is the effect caused by the overexpression of miR-143-3p, observed in podocytes, which, when stimulated by TGF-β, showed an increase in this miRNA’s expression in response to the stimulus [26,27]. This upregulation resulted in the negative regulation of glycoproteins such as syndecan (SDC) and versican (VCAN) [26]. These glycoproteins are involved in intercellular adhesion, migration, proliferation, and cellular differentiation processes [28].

According to Müller-Deile and colleagues [26], the downregulation of these proteins resulted in alterations in the structure and function of the glomerular filtration barrier, contributing to the development of a nephrotic profile in zebrafish larvae. This nephrotic profile was mainly characterized by the loss of plasma proteins, observed through fluorescence detected in the ocular vessels of Tg(l-fabp:DBP:EGFP) zebrafish under optical microscopy [26]. Additionally, there may also be a paracrine crosstalk between podocytes and other cells of the renal structure, such as Glomerular Endothelial Cells (GECs), through the release of Exosomal Vesicles (EVs) containing miRNAs excreted by podocytes [17].

This type of communication has been previously observed between GECs and other cell types [29]. Another indication of this communication is that the increase in miR-143 expression in podocytes results in the negative regulation of VCAN and SDC isoforms not only in podocytes but also in glomerular endothelial cells, since these cells also express isoforms of miR-143 target genes [26], such as SDC1, SDC3, and VCAN, and since these cells are histologically adjacent to podocytes [30], they may be subject to regulation by interference from external EVs. In summary, these data indicate that glomerular glycocalyx proteins (DSC and VCAN) are regulated by miR-143 and that miR-143 may be a novel agent in TGF-β-induced glomerulonephropathy, as its overexpression causes functional and structural impairments in the glomerular filtration barrier [26].

Currently, there is not much information in the global literature regarding miR-143-3p and Diabetic Nephropathy. In a previous study made by Perez-Hernandez et al. [17], individuals with hypertension who also had DKD showed an upregulation of miR-143-3p, particularly in the urine exosome miRNome. In that study, miR-143-3p was one of the miRNAs selected for validation in a confirmatory group of hypertense patients with and without albuminuria. However, the results showed no significant differences in miR-143-3p expression levels between the groups [16].

Regarding DKD, miR-143-3p may be involved in its pathogenesis, as in a study by Müller-Deile and collaborators, where the overexpression of miR-143 results in a nephrotic phenotype, including generalized edema, loss of plasma proteins, the swelling of glomerular endothelial cells and the loss of glomerular endothelial fenestration. These data indicate a dysfunction in the glomerular filtration barrier [24].

### 3.3. hsa-miR-501-3p

Although significant differences in expression levels between the groups (Non vs. UAE) in our cohort for miR-501-3p were not found, previous studies highlight variations in this miRNA’s levels. For example, in the Chinese population, DKD patients in stage V presented a significant downregulation of miR-501-3p when compared with DKD patients in stage I [18]. In in vitro cells, the overexpression of mir-501-3p markedly inhibits cellular proliferation, inducing an interruption of the G1 phase [31]. This mechanism happens through the genetic suppression of WTAP, a target gene of mir-501-3p, the data of which were confirmed by other experiments. The WTAP suppression produces inhibitor effects of cellular proliferation [31].

According to the literature, miR-501-3p was identified as differentially expressed (downregulated) in serum samples from patients with Alzheimer’s disease [32] and breast cancer cells [33]. Thus, miR-501-3p seems to be intimately involved in cell proliferation [32,33]. Cell proliferation is commonly observed in the early stages of DKD, in which there is an increase in GFR because of an increase of glomerular mass, in response to incentives, such as induction by TGF-β [25]. With its dual behavior in disorders related to cell proliferation, it is not possible to precisely determine whether miR-501-3p acts in a pathogenic or protective manner, as it may be attempting to suppress a pathogenic biological process as well as contributing to the development of this pathology.

### 3.4. hsa-miR-100-5p

Here, we found that hsa-miR-100-5p may be associated with nephropathy protection. Assmann et al. indicate a lower miR-100-5p expression in patients with T1DM without comorbidities [19]. However, we did not identify any suggestions of an involvement of this miRNA in albuminuria in patients with T1DM, so this finding is the first related to the association of this miRNA with Diabetic Nephropathy. This review revealed that miR-100 is significantly downregulated in serum/plasma samples from patients with T1DM [19].

In addition, other authors have identified that, through the interaction with the mechanistic Target of Rapamycin (mTOR) signaling pathway, this miRNA may be involved in cell growth and proliferation [19,34,35]. mTOR is a crucial protein kinase in regulating cell growth and proliferation, as well as integrating signals from nutrients, energy, and growth factors to coordinate the cellular response. It plays a crucial role in regulating protein biosynthesis, ribosome biogenesis, and protein translation, promoting cell growth. In addition, mTOR plays an important role in regulating mitophagy. Under favorable conditions, such as high nutrient and energy availability, mTORC1 inhibits mitophagy by suppressing the formation of autosomes surrounding damaged mitochondria for degradation. On the other hand, in situations of cellular stress, such as oxidative stress, the inhibition of mTORC1 allows for the activation of mitophagy to remove dysfunctional mitochondria, suppressing it in favorable conditions and allowing it to maintain cellular homeostasis [36].

Considering our data, and the previous discussion, a reduced expression of miR-100 in patients with MIC was observed when compared to non-UEA. Hence, we hypothesize that the downregulation of miR-100-5p may represent a protective response against the various pathological processes observed in the early stages of DKD, since the underexpression of that miRNA can be a molecular answer to DKD development. Thus, this allows for the normal function of pathways in which target genes act, resulting in a protective cellular mechanism that tries to combat DKD progress. Some modifications produced by DKD are glomerular hypertrophy and mesangial expansion, which are directly related to cell growth and proliferation, which contribute, for example, to the increase of GFR. The increase in the glycation process is another phenomenon observed at the onset of DM which intensifies in kidney cells as DKD progresses. This provokes an increase in Advanced Glycation End-products (AGEs) inside the cells. All these previous processes result, directly or indirectly, in the cell damage and activation of immune and mitochondrial mechanisms [37]. However, further research is needed to elucidate the miR-100 behavior in DKD.

The same was not observed when comparing the mean ΔCT of miR-100-5p between the MIC and Non-UAE groups, where we observed an increase in albuminuria excretion and a decrease in GFR, due to the increase in endothelial fenestral space and mainly renal functional cell death.

### 3.5. Target Genes

**Figure 3:**
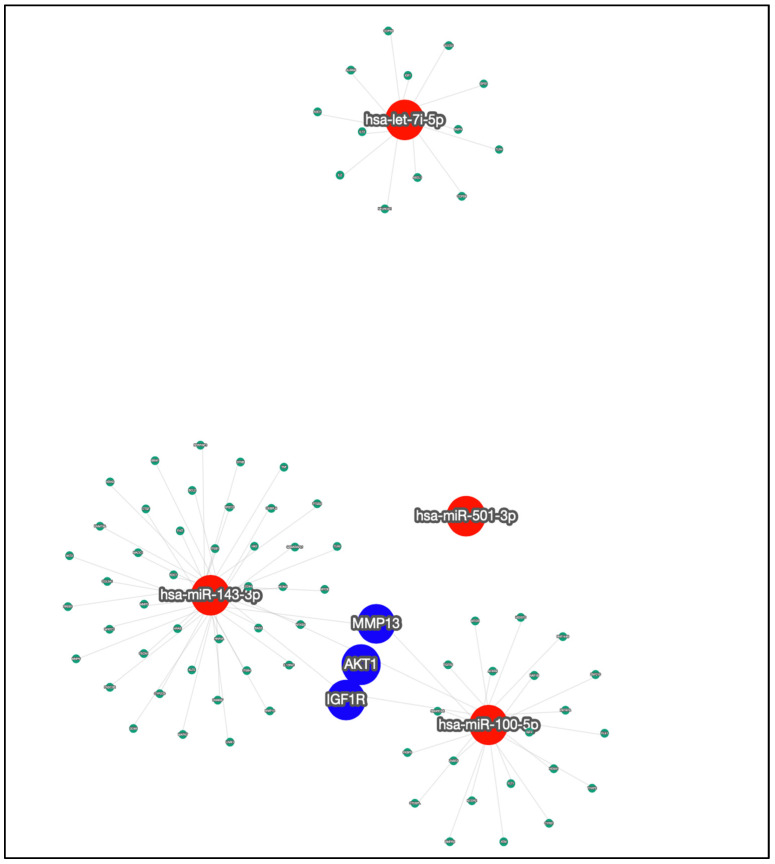
Network association between the four miRNAs and their target genes. The investigated miRNAs are in red and their target genes are in green, except for the three genes in blue, which are the target genes in the intersection of two miRNAs.

We used miRTargetLink 2.0 [38] to investigate the target genes related to the four miRNAs previously analyzed and selected just the relevant ones. The relevance criteria follow miRTarbase, which considers Reporter assay, Western blot and qPCR as strong validators, and microarray, NGS, pSILAC, CLIP-Seq and other methods as less strong validators [38]. We observed that three genes are linked to both miR-100-5p and miR-143-3p, which are IGF1R, AKT1, and MMP13. Figure 3 and Supplementary Table S1 show this information.

The *IGF1R* gene (Insulin like-Growth Factor Receptor), develops a crucial role in metabolic actions and has autocrine, endocrine, and paracrine functions. It has been shown that IGF1R inhibition can annulate some DKD symptoms, such as albuminuria in mice [34], following previous studies [39]. Moreover, AKT1 is also a target gene that is related to some important pathways, such as insulin resistance, PI3K-Akt signaling, TGF-β signaling, MAPK signaling, the insulin signaling pathway, TNF signaling, and AGE-RAGE signaling in diabetic complications [40]. Finally, MMP13 overexpression can promote renal tubular epithelial cell injury [41], possibly because the MMP, a family of zinc-dependent endoproteases, is generally responsible for some metabolic processes as well, such as apoptosis, angiogenesis, and tissue regeneration [42].

## 4. Materials and Methods

### 4.1. Sampling and Ethical Aspects

In this research, 20 individuals with diagnosed T1DM were included, according to the criteria from the American Diabetes Association (ADA) [43]. Urinary Albumin Excretion (UAE) over 24 h was used to measure albuminuria in the selected samples. Albuminuria was considered if two out of three consecutive measures were high.

The categories used for classification were UAE not elevated (Non) (n = 13) for values up to 30 mg/24 h; microalbuminuria (MIC) (n = 4) for values higher than 30 mg/24 h; and macroalbuminuria (MAC) (n = 3), for values equal to or higher than 299 mg/24 h. The Research Ethics Committee of the João de Barros Barreto University Hospital (HUJBB, Belém, Pará, Brazil) approved this work (n. 005/12). All procedures involving human participants followed the ethical guidelines of the Declaration of Helsinki. All participants gave written informed consent.

### 4.2. Selection of miRNAs

The expression data of hsa-let-7i-5p, hsa-miR-143-3p, hsa-miR-501-3p, and hsa-miR-100-5p were obtained from the previous study made by Ferraz et al. [15]. These miRNAs were selected because they had the highest fold change and the lowest *p*-value. The original work used RT-qPCR for validation, following protocols of RNA extraction, quantification, and amplification.

### 4.3. Data Analysis

The expression levels of the studied miRNAs were normalized using the comparative Ct method [44] and miR-16 was used as an endogenous control. *T*-tests or Mann–Whitney tests were used when comparing two groups if the data were parametric or non-parametric, respectively. If three or more groups were compared, we used ANOVA or Kruskal–Wallis followed by pairwise comparisons adjusted by the false discovery rate (FDR) method. All statistical analyses and graphs were performed using R v.4.0.2 and *p*-values < 0.05 were considered statistically significant.

## 5. Conclusions

In conclusion, our study highlights the potential role of hsa-miRNA-100-5p in protecting against nephropathy in T1DM, as indicated by its decreased expression in patients with microalbuminuria. While hsa-miR-501-3p and hsa-miR-143-3p have been extensively studied in different conditions, our analysis did not find significant differences in their expression levels between the groups. Our findings also suggest a regulatory role of miRNAs in modulating target genes such as *AKT1*, *MMP13*, and *IGF1R*, implicating them in signaling pathways and cellular metabolism relevant to Diabetic Kidney Disease.

## Figures and Tables

**Table 1:** Clinical characteristics of the different T1DM groups.

Variable	Non (n = 12)	MIC (n = 4)	MAC (n = 3)
BMI	23.1 ± 3.9	22.7 ± 2.2	29.8 ± 6.6
FBG	203.2 ± 101.3	76.3 ± 27.5	136.7 ± 23.4
GFR	121.8 ± 12.1	117.1 ± 26.5	83.7 ± 33.4
Creatinine	0.7 ± 0.2	0.8 ± 0.3	1.4 ± 0.2

Data are expressed as mean ± SD. BMI indicates Body Mass Index; FBG, Fasting Blood Glucose; and GFR, Glomerular Filtration Rate. FBG with multiple significant comparisons: MIC vs. Non (*p* = 0.036); GFR, MAC vs. Non (*p* = 0.019); Creatinine, MAC vs. MIC (*p* = 0.001) and MAC vs. Non (*p* < 0.001); Urea, MAC vs. Non (*p* = 0.016). No difference was observed in BMI between the groups (*p* = 0.052).

## Data Availability

The original data is available from doi:10.3389/fendo.2022.1033809.

## Supplementary Materials

The supporting information can be downloaded at: https://www.mdpi.com/article/10.3390/ijms25115663/s1.
